# Enhanced YB1/EphA2 axis signaling promotes acquired resistance to sunitinib and metastatic potential in renal cell carcinoma

**DOI:** 10.1038/s41388-020-01409-6

**Published:** 2020-08-19

**Authors:** Hailong Ruan, Sen Li, Lin Bao, Xiaoping Zhang

**Affiliations:** 1 Department of Urology, Union Hospital, Tongji Medical College, Huazhong University of Science and Technology, Wuhan, 430022 China; 2 Institute of Urology, Union Hospital, Tongji Medical College, Huazhong University of Science and Technology, Wuhan, 430022 China

**Keywords:** Renal cell carcinoma, Cell migration

## Abstract

VHL mutations are the most common tumorigenic lesions in clear cell renal cell carcinoma (ccRCC) and result in continued activation of the HIF/VEGF pathway and uncontrolled cancer progression. Receptor tyrosine kinase (RTK) inhibitors such as sunitinib have been demonstrated to target tumorigenic signaling pathways, delay tumor progression, and improve patient prognosis in metastatic renal cell carcinoma (mRCC). Although several mechanisms of sunitinib resistance have been reported, the solutions to overcome this resistance remain unclear. In our study, we found that increased expression of Y-box binding protein 1 (YB1, a multidrug resistance associated protein) and EphA2 (a member of the erythropoietin-producing hepatocellular (Eph) receptor family, belonging to the RTK family) mediated sunitinib resistance and mRCC exhibited a large phenotypic dependence on YB1 and EphA2. In addition, our findings confirm that YB1 promotes the invasion, metastasis and sunitinib resistance of ccRCC by regulating the EphA2 signaling pathway. Furthermore, pharmacological inhibition of EphA2 through the small molecule inhibitor ALW-II-41-27 reduced the proliferation of sunitinib-resistant tumor cells, suppressed tumor growth in vivo, and restored the sensitivity of sunitinib-resistant tumor cells to sunitinib in vitro and in vivo. Mechanistically, YB1 increases the protein levels of EphA2 by maintaining the protein stability of EphA2 through inhibition of the proteasomal degradation pathway. Collectively, our findings provide the theoretical rationale that ccRCC metastasis and RTK-directed therapeutic resistance could be prospectively and purposefully targeted.

## Introduction

Clear cell renal cell carcinoma (ccRCC), the most prevalent subtype of renal cell carcinoma (RCC), is characterized by VHL mutations in 70–80% of cases, high metastasis rate and mortality, and resistance to radiotherapy and chemotherapy. It is estimated that more than 90% of cancer-related deaths are attributed to tumor metastasis [1]. The abundant vascularization surrounding ccRCC tissue provides a convenient medium for tumor spread and metastasis [2]. Although great progress has been made in recent years on the molecular mechanism of ccRCC metastasis, the crucial mechanism driving ccRCC metastasis remains unclear. Receptor tyrosine kinase (RTK) inhibitors such as sunitinib (SUN) have been approved for the first-line treatment of metastatic RCC (mRCC) [3, 4]. However, many patients with mRCC are unable to use such molecular-targeted drugs due to the presence of primary or acquired drug resistance [5]. Although several mechanisms of SUN resistance have been reported [6, 7], the specific solutions remain unclear. Therefore, the mechanisms of ccRCC metastasis and SUN resistance need to be urgently defined for the clinical development of new therapeutic targets.

Y-box binding protein 1 B1 (YB1), a member of the cold-shock domain protein family, has been reported to be highly expressed in a variety of malignancies including renal cancer [8–11]. As a DNA-binding protein, YB1 can increase the transcription of many genes, such as cyclin A [12] and topoisomerase IIa [13]. As an RNA-binding protein, YB1 can bind snail1 and HIF1α mRNA to activate their translation and promote tumor metastasis [14, 15]. Our group and others have confirmed that, in other malignancies, YB1 mainly functions to promote cancer metastasis and multidrug resistance [16, 17]. A previous study reported that YB1 is highly expressed in renal cancer and promotes tumor metastasis [11]. However, the crucial roles of YB1 in SUN resistance and the mechanisms by which YB1 promotes ccRCC metastasis have not been studied.

EphA2 (a member of the erythropoietin-producing hepatocellular (Eph) receptor family, belonging to the RTK family) is one of the most studied members of the Eph receptor family. In melanoma, EphA2 is overexpressed and mediates vemurafenib resistance [18]. EphA2 blockade was reported to overcome resistance to EGFR kinase inhibitors in lung cancer [19]. High EphA2 levels are correlated with poor prognosis in oesophageal cancers and glioblastomas and promote the proliferation and metastasis of various malignant tumor cells [20, 21]. Our previous study reported that EphA2 is highly expressed in ccRCC and promotes the migration and invasion of ccRCC cells [22]. High levels of EphA2 expression are correlated with tumor grade, increased vascularization, and poor overall survival (OS) in RCC [23]. The above studies show that EphA2 plays a crucial role in tumor metastasis and drug resistance. However, the mechanisms by which EphA2 plays a role in SUN resistance and EphA2 is overexpressed in ccRCC remain unclear.

In our study, we demonstrate for the first time that YB1 and EphA2 are highly expressed in SUN-resistant ccRCC cell lines and that the SUN-resistant phenotype is significantly dependent on high levels of YB1 and EphA2 expression. In addition, we found that YB1 could promote the invasion, metastasis, and SUN resistance of ccRCC, which could be neutralized by knockdown of EphA2. Furthermore, pharmacological inhibition of EphA2 by the small molecule inhibitor ALW-II-41-27 reduced the proliferation of SUN-resistant tumor cells, suppressed tumor growth in vivo, and restored the sensitivity of SUN-resistant tumor cells to SUN in vitro and in vivo. Mechanistically, YB1 increases the protein levels of EphA2 by maintaining the protein stability of EphA2 through inhibition of the proteasomal degradation pathway. These results demonstrated that YB1 and EphA2 might play roles as oncogenes and drug-resistant genes in the progression of ccRCC and provided a rationale for consideration of the combined treatments of YB1 and EphA2 as a novel therapeutic strategy for metastatic and SUN-resistant ccRCC.

## Results

### YB1 is highly expressed and positively correlated with epithelial–mesenchymal transition (EMT) in ccRCC

**Fig. 1:**
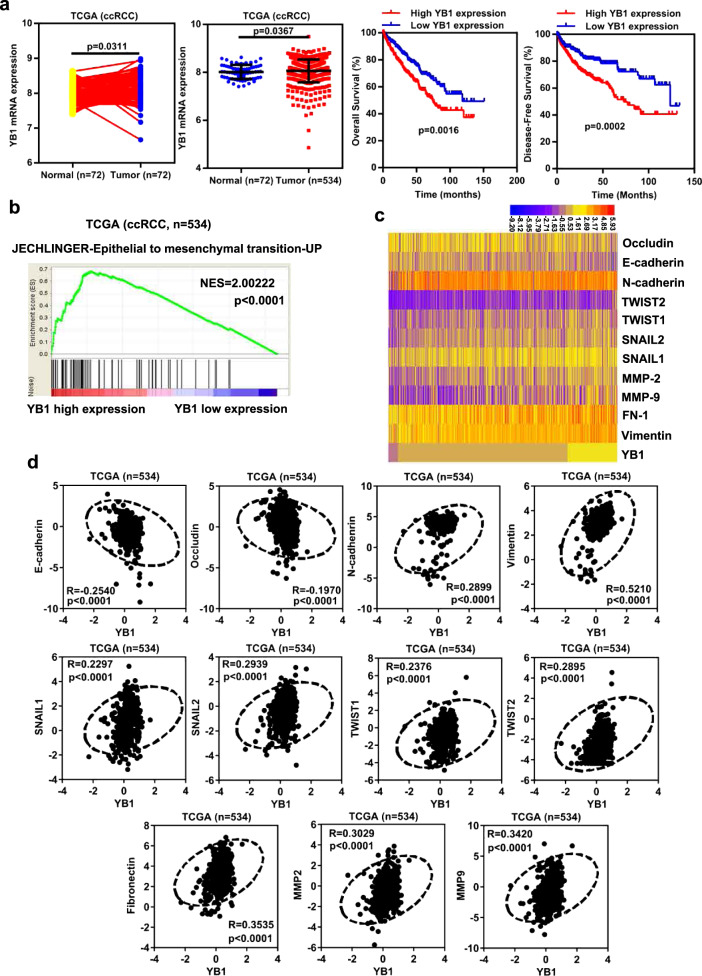
YB1 is highly expressed and positively correlated with epithelial–mesenchymal transition (EMT) in ccRCC. **a** YB1 mRNA levels were significantly upregulated in a TCGA dataset composed of 534 ccRCC tissues, including 72 paired tissues. Kaplan–Meier analysis of YB1 in ccRCC patients for OS and DFS. **b** GSEA analysis between YB1 mRNA levels and EMT signaling pathways. **c** Heat map depicting the association of YB1 with epithelial markers and mesenchymal markers. **d** Correlation analysis between YB1 and EMT markers.

We first evaluated the expression of YB1 in ccRCC cancer tissues and adjacent normal tissues using the publicly available TCGA database. As shown in Fig. 1a, YB1 expression levels were higher in cancer tissues than in adjacent normal tissues, and high YB1 expression correlated with poor OS and disease-free survival (DFS). A previous study reported that YB1 promotes EMT and tumor metastases in breast cancer [14]. Thus, we analyzed the correlation between YB1 expression and EMT marker expression in ccRCC. Gene set enrichment analysis (GSEA) performed using the ccRCC gene pool in the TCGA database showed that genes regulated by high YB1 expression were mainly concentrated in the EMT pathway (Fig. 1b) [24]. Heat map and correlation analysis indicated that the expression of YB1 was positively correlated with the expression of mesenchymal markers (N-cadherin, vimentin, Snail1, Snail2, Twist1, Twist2, fibronectin, MMP2, and MMP9), but negatively correlated with the expression of epithelial markers (E-cadherin and occludin) (Fig. 1c, d). These results indicated that YB1 was highly expressed and might promote EMT in ccRCC.

### The expression of YB1 correlates with a wide range of clinicopathological parameters in ccRCC

To assess whether YB1 expression is correlated with clinicopathological parameters, we evaluated the correlation between YB1 expression and clinicopathological parameters (Table 1). After analyzing the TCGA data, we found no correlation between YB1 expression and patient age (Supplementary Fig. 1a), but YB1 expression was higher in male patients than in female patients (Supplementary Fig. 1b). High YB1 expression levels were also significantly associated with higher TNM stage, T stage, M stage, G stage, and poorer OS and DFS (Supplementary Fig. 1c, d, f–i). Surprisingly, the level of YB1 expression was not associated with lymph node metastasis (Supplementary Fig. 1e). The above data showed that the expression level of YB1 was correlated with various clinicopathological parameters in ccRCC.

**Table 1:** Correlation between YB1 mRNA expression and clinicopathological parameters of ccRCC patients.

Parameter	Number	YB1 mRNA expression		*P* value
		Low (*n* = 124)	High (*n* = 124)	
Age (years)				1
≥60	144	72	72	
<60	104	52	52	
Gender				0.0001
Male	151	61	90	
Female	97	63	34	
T stage				0.0098
T1 + T2	146	83	63	
T3 + T4	102	41	61	
Lymph node metastasis				0.0165
N0	233	121	112	
N1	15	3	12	
M stage				0.0262
M0	207	110	97	
M1	41	14	27	
Histologic grade				0.0014
G1 + G2	111	68	43	
G3 + G4	137	56	81	
Pathologic stage				0.0009
p I + II	134	80	54	
p III + IV	114	44	70	

### YB1 accelerates the mobility of human renal cancer cells in vitro

**Fig. 2:**
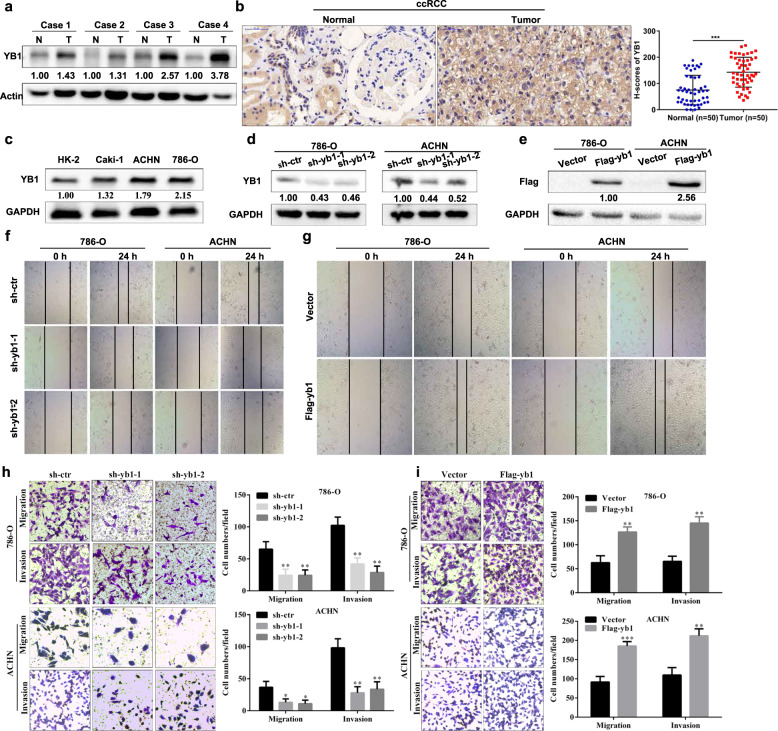
YB1 accelerates the mobility of human renal cancer cells in vitro. **a**, **b** Western blot and immunohistochemical analysis of YB1 expression in ccRCC tissues (N normal tissues, T tumor tissues). *H*-scores of YB1 immunohistochemical staining were presented. The values indicate protein expression levels relative to actin levels. **c** Western blot analysis of YB1 expression in renal normal epithelial cells (HK-2) and tumor cells (Caki-1, ACHN, 786-O). The values indicate protein expression levels relative to GAPDH levels. **d**, **e** Western blot validation of stable YB1 knockdown or overexpression in the indicated cell lines. The values indicate protein expression levels relative to GAPDH levels. **f**, **g** Scratch experimental analysis of the effect of stable YB1 knockdown or overexpression on cell migration. **h**, **i** Transwell experimental analysis of the effect of stable YB1 knockdown or overexpression on cell migration and invasion (***P < 0.001, **P < 0.01, *P < 0.05, compared with the corresponding control).

To further validate the TCGA cancer database results, ccRCC tissues were subjected to western blotting and immunohistochemistry (IHC) analysis (Fig. 2a, b). All results confirmed that YB1 expression in ccRCC tissues was higher than that in corresponding normal tissues, consistent with the results of the TCGA database analysis. Next, we examined the expression of YB1 in renal cancer cell lines and found that the increased expression of YB1 in tumor cells was consistent with the results of the ccRCC tissues (Fig. 2c). Since YB1 was positively correlated with EMT, we speculated that YB1 might promote cell migration and invasion in ccRCC. We successfully constructed 786-O and ACHN cell lines with stable YB1 knockdown or stable YB1 overexpression (Fig. 2d, e). Cell scratch assay results showed that the knockdown of YB1 expression inhibited cell migration, while the overexpression of YB1 accelerated cell migration (Fig. 2f, g). In line with our expectations, the results of the transwell migration and invasion experiments were consistent with the experimental results of the scratch assays (Fig. 2h, i). To investigate the effect of VHL on the YB1/EphA2 axis, we also included Caki-1 cells, a cell line identified as VHL wild type, in our experiments. As shown in Supplementary Fig. 2a, VHL knockdown did not affect the expression of YB1 or EphA2 in Caki-1 cells. Moreover, stable knockdown or overexpression of YB1 in Caki-1 cells resulted in a phenotype similar to that of 786-O and ACHN cells (Supplementary Fig. 2b, c). These findings indicated that YB1 promoted the migration and invasion of renal cancer cells in vitro.

### YB1 positively regulates tumor metastasis and drug resistance signaling pathways

**Fig. 3:**
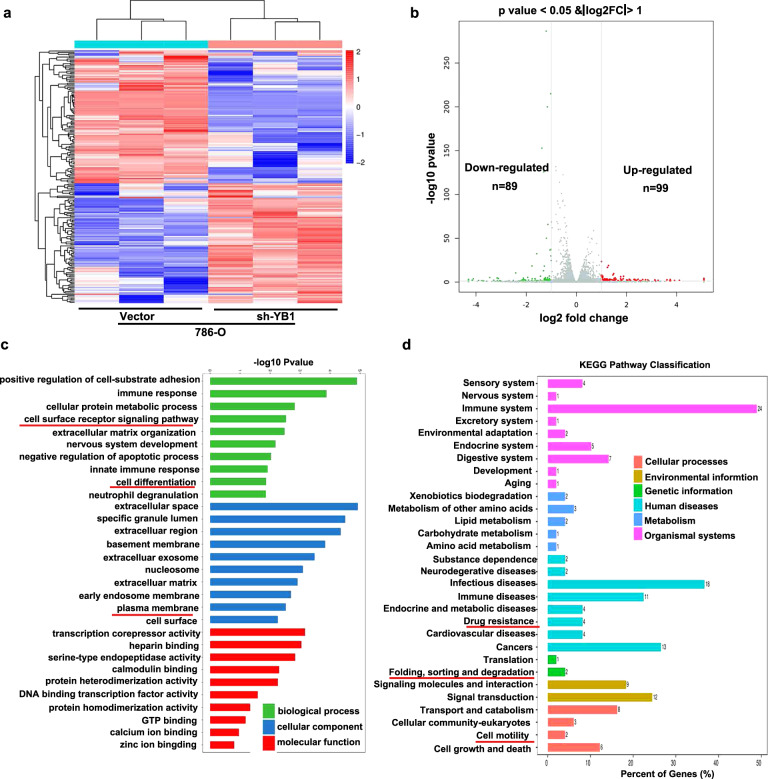
YB1 positively regulates tumor metastasis and drug resistance signaling pathways. **a** Heat map of mRNA sequencing results in YB1 knockdown and control cells. **b** Volcano map reveals up- and downregulated genes in 786-O cells with YB1 knockdown. **c** GO analysis showed that genes downregulated by YB1 knockdown were involved in the cell surface receptor signaling pathway. **d** KEGG analysis of genes downregulated by YB1 knockdown.

The positive correlation between YB1, metastasis, and EMT signaling pathways suggests that YB1 may function via these pathways. To validate this hypothesis, we performed high-throughput mRNA sequencing in 786-O cells with stable YB1 knockdown. The results of high-throughput sequencing are plotted as a heat map (Fig. 3a). Volcano plots show upregulated and downregulated genes in 786-O cells with YB1 knockdown according to the screening criteria (Fig. 3b). Subsequently, GO analysis revealed that genes downregulated by YB1 knockdown were involved in the cell surface receptor signaling pathway (Fig. 3c). KEGG enrichment analysis indicated that the downregulated genes in YB1 knockdown cells were enriched in multiple signaling pathways, such as drug resistance, protein folding and degradation, cell motility, cell growth, and cell death (Fig. 3d). Collectively, these findings suggested that YB1 might positively regulate tumor metastasis and drug resistance signaling pathways.

### YB1 is positively correlated with the expression of EphA2 and can significantly rescue the EphA2 knockdown phenotype

**Fig. 4:**
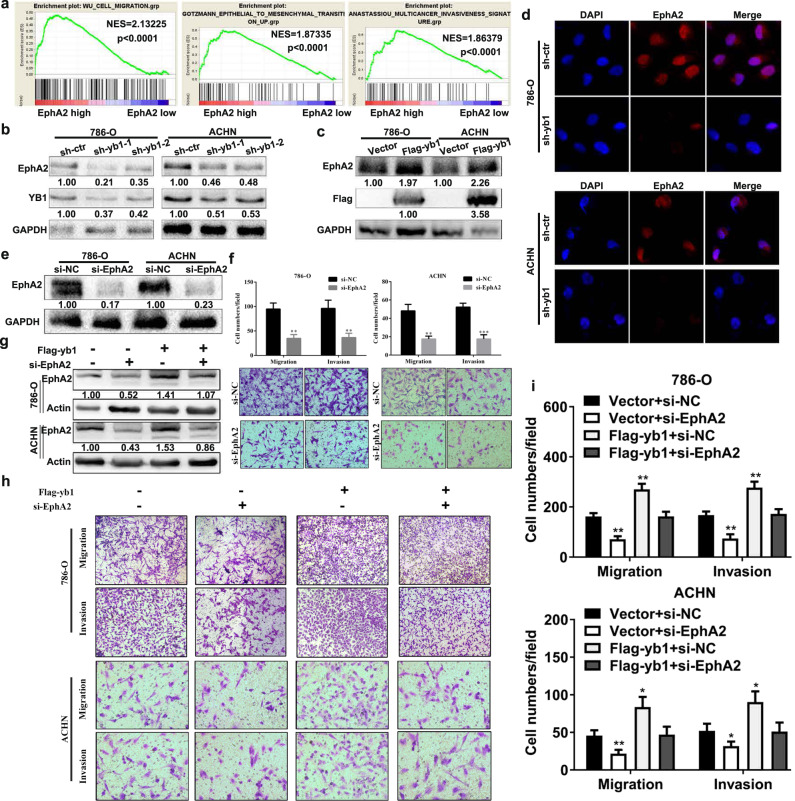
The effect of EphA2 expression on tumor cell migration and invasion. **a** GSEA analysis between EphA2 mRNA levels and metastasis-related signaling pathways. **b**, **c** Western blot analysis of YB1 and EphA2 proteins in 786-O and ACHN cells with YB1 knockdown or overexpression. The values indicate protein expression levels relative to GAPDH levels. **d** Immunofluorescence analysis of the EphA2 protein in 786-O and ACHN cells with YB1 knockdown. **e**, **f** Transwell analysis of the effect of EphA2 knockdown on renal cancer cell migration and invasion. The values indicate protein expression levels relative to GAPDH levels. **g** Western blot analysis of YB1 and EphA2 protein expression in 786-O and ACHN cells with YB1 overexpression/EphA2 knockdown or vector/EphA2 knockdown. The values indicate protein expression levels relative to GAPDH levels. **h**, **i** Transwell analysis of the migration and invasion capability of 786-O and ACHN cells with YB1 overexpression/EphA2 knockdown or vector/EphA2 knockdown (***P < 0.001, **P < 0.01, *P < 0.05, compared with the corresponding control).

Our previous study found that EphA2 expression was elevated and that high EphA2 expression contributed to cell migration and invasion in renal cancer [22]. Our findings indicated that both YB1 and EphA2 could promote the invasion and metastasis of renal cancer cells, but the regulatory network between them remained unclear. Our RNA-sequencing results showed that genes downregulated by YB1 knockdown were involved in the cell surface receptor signaling pathway (Fig. 3c), and EphA2 is a key molecule in the cell surface receptor signaling pathway. The above results indicate the possibility that YB1 may be an upstream regulatory element of EphA2. To understand the specific relationship between YB1 and EphA2, we performed immunohistochemical staining of YB1 and EphA2 using tissue microarrays (TMAs) with 50 pairs of ccRCC tissues. TMA staining results showed that both YB1 and EphA2 were upregulated in ccRCC, and YB1 was positively correlated with EphA2 in tumor tissues (*r* = 0.3374, *P* = 0.0166) (Supplementary Fig. 3a). Moreover, by analyzing the TCGA database, we found that EphA2 is highly expressed and correlated with various clinicopathological parameters in ccRCC, including gender, T stage, G stage, and TNM stage (Table 2 and Supplementary Fig. 4a–h). GSEA analysis showed that genes regulated by high EphA2 were enriched in cell migration, EMT, and multicancer invasiveness signature signaling pathways (Fig. 4a). Therefore, we analyzed the relationship between EphA2 and EMT markers using the TCGA database and found that EphA2 was positively correlated with mesenchymal markers, including N-cadherin, vimentin, SNAIL1, MMP2, TWIST1, and TWIST2 (Supplementary Fig. 5a). Subsequently, we demonstrated that knockdown or overexpression of YB1 could downregulate or upregulate EphA2 expression in human renal cancer cells, respectively (Fig. 4b–d). Knockdown of EphA2 expression inhibited cell migration and invasion in human renal cancer cells (Fig. 4e, f). The above results raised the possibility that YB1 might directly or indirectly regulate EphA2 expression. To test this possibility, we performed YB1 rescue experiments in EphA2 knockdown cells and corresponding control cells. As shown in Fig. 4g–i, YB1 not only restored EphA2 protein expression but also rescued the metastatic phenotype of EphA2 knockdown in human renal cancer cells. In addition, we observed a similar rescue phenotype in Caki-1 cells (Supplementary Fig. 2d). These results suggested that YB1 regulated the migration and invasion of renal cancer cells via EphA2 signaling.

**Table 2:** Correlation between EphA2 mRNA expression and clinicopathological parameters of ccRCC patients.

Parameter	Number	EphA2 mRNA expression		*P* value
		Low (*n* = 260)	High (*n* = 261)	
Age (years)				
<60	241	116	125	
≥60	280	144	136	0.453
Gender				
Male	339	182	157	
Female	182	78	104	0.018
T stage				
T1 + T2	333	153	180	
T3 + T4	188	107	81	0.016
N stage				
N0 + NX	506	251	255	
N1	15	9	6	0.427
M stage				
M0 + MX	443	216	227	
M1	78	44	34	0.213
G stage				
G1 + G2	241	105	136	
G3 + G4	280	155	125	0.007
TNM stage				
I + II	315	144	171	
III + IV	206	116	90	0.018

### YB1 maintains the protein stability of EphA2 by inhibiting the proteasome degradation pathway

**Fig. 5:**
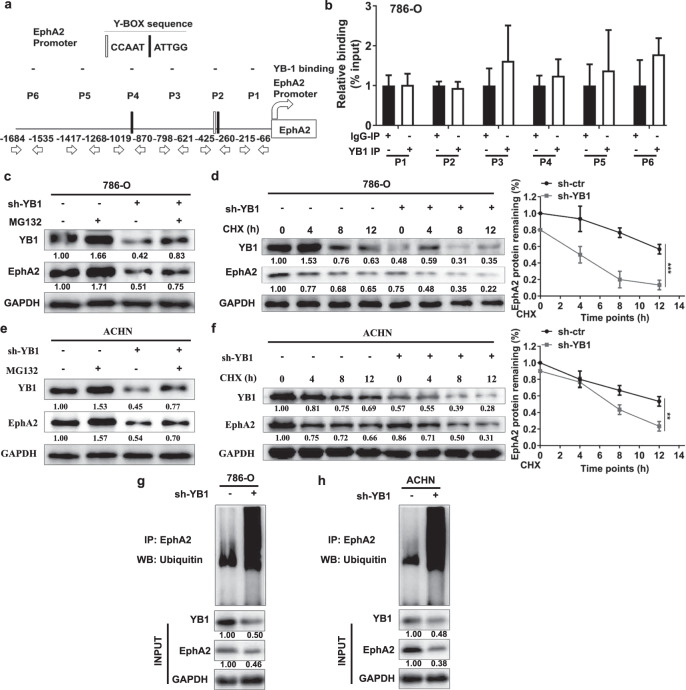
YB1 maintains the protein stability of EphA2 by inhibiting the proteasome degradation pathway. **a** The human EphA2 proximal promoter region [−1 to −2 kb upstream of the transcription start site (TSS)] had three Y-Box sequences (CCAAT/ATTGG). **b** ChIP analysis of YB1 for the EphA2 promoter region in 786-O cells. ChIP assays were performed using anti-IgG and anti-YB1 antibodies. **c** EphA2 protein accumulation was analyzed after treatment with MG132 in YB1 knockdown 786-O cells. The values indicate protein expression levels relative to GAPDH levels. **d** After cycloheximide (CHX) treatment, the half-life of EphA2 protein was analyzed in 786-O cells (vector and shYB1). The values indicate protein expression levels relative to GAPDH levels. **e** EphA2 protein accumulation was analyzed after treatment with MG132 in YB1 knockdown ACHN cells. The values indicate protein expression levels relative to GAPDH levels. **f** After cycloheximide (CHX) treatment, the half-life of EphA2 protein was analyzed in ACHN cells (vector and shYB1). The values indicate protein expression levels relative to GAPDH levels. **g**, **h** Co-IP analysis of ubiquitination modification of EphA2 in 786-O and ACHN cells (vector and shYB1). The values indicate protein expression levels relative to GAPDH levels. (***P < 0.001, **P < 0.01, compared with the corresponding control).

Our previously mentioned results suggested that YB1 could regulate EphA2 expression and rescue the EphA2 knockdown phenotype. However, the specific mechanism by which YB1 regulates EphA2 remains unknown. To solve this problem, we first searched the promoter region of EphA2 and found that the EphA2 proximal promoter region (−1 to −2000 kb upstream of the transcription start site) contained three Y-Box putative binding sequences (ATTGG/CCAAT) (Fig. 5a). Next, the EphA2 proximal promoter region was divided into six promoter fragments (P1–P6, as indicated). Then, chromatin immunoprecipitation (ChIP) experiments were performed in 786-O cells and the results showed that YB1 could not bind to any promoter fragment, including the fragments containing the Y-Box sequences (Fig. 5b). Moreover, our high-throughput sequencing results confirmed that knockdown of YB1 did not significantly change the levels of EphA2 mRNA (data not shown). In addition, the results of TMA staining confirmed a positive correlation between YB1 and EphA2 protein expression in ccRCC tissues (Supplementary Fig. 3a). These results showed that YB1 could not regulate EphA2 expression at the transcriptional level, suggesting that YB1 might control EphA2 expression by regulating the stability of the EphA2 protein.

We used the proteasome inhibitor MG132 to explore the mechanisms of YB1-dependent EphA2 protein stabilization. Treatment of YB1 knockdown 786-O and ACHN cells with MG132 increased EphA2 protein accumulation (Fig. 5c, e), indicating constitutive EphA2 degradation in YB1 knockdown 786-O and ACHN cells. Accordingly, we used cycloheximide to inhibit protein synthesis in 786-O and ACHN cells with stable YB1 knockdown, and EphA2 protein turnover was examined over time. Compared with that of the corresponding control cells, the half-life of EphA2 was dramatically reduced in 786-O and ACHN cells with stable YB1 knockdown (Fig. 5d, f). Furthermore, we also explored the effect of YB1 on the ubiquitin modification of EphA2 protein, and the experimental results showed that YB1 knockdown increased the ubiquitin modification of EphA2 protein in 786-O and ACHN cells (Fig. 5g, h). Taken together, these findings indicated that YB1 maintained the stability of EphA2 protein by inhibiting the proteasome degradation pathway.

### YB1 induces SUN resistance by mediating EphA2 signaling in vitro

**Fig. 6:**
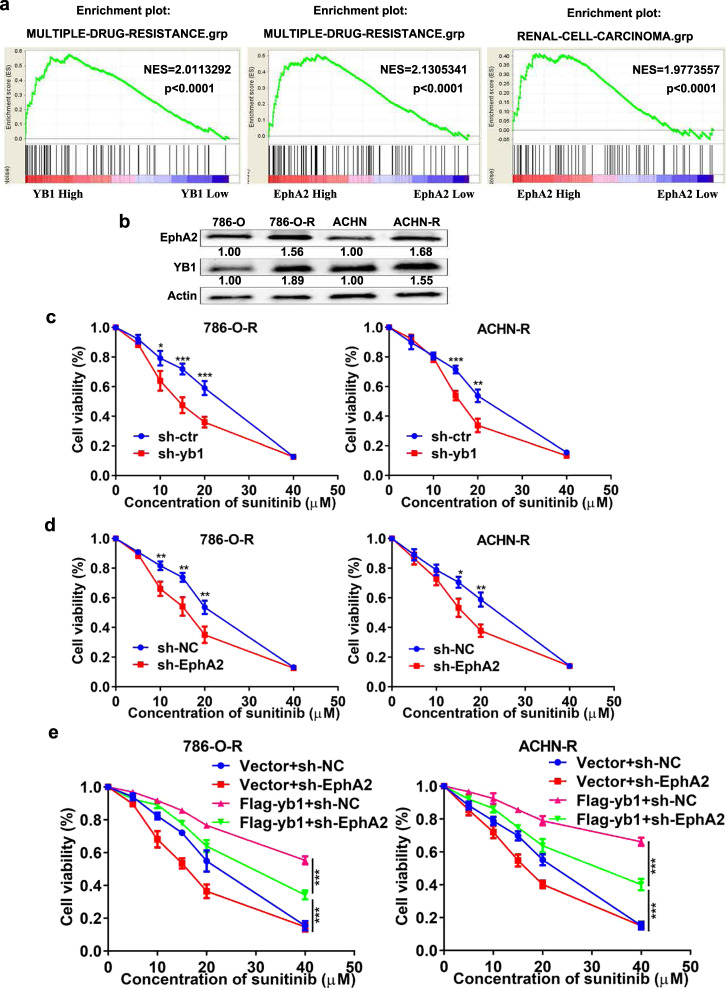
YB1 induces sunitinib resistance by mediating EphA2 signaling in vitro. **a** GSEA analysis between YB1 and EphA2 mRNA levels and multiple-drug resistance signaling pathways. **b** Western blot analysis of YB1 and EphA2 protein expression in parental and sunitinib-resistant cells. The values indicate protein expression levels relative to actin levels. **c** CCK8 assay analysis for the effect of YB1 knockdown on the sensitivity of sunitinib-resistant cells to sunitinib treatment. **d** CCK8 assay analysis for the effect of EphA2 knockdown on the sensitivity of sunitinib-resistant cells to sunitinib treatment. **e** CCK8 assay analysis for the sensitivity to sunitinib treatment of 786-O-R and ACHN-R cells with YB1 overexpression/EphA2 knockdown or vector/EphA2 knockdown (***P < 0.001, **P < 0.01, *P < 0.05, compared with the corresponding control).

KEGG analysis of our RNA-sequencing results showed that YB1-mediated genes could be enriched in drug resistance signaling pathways (Fig. 3d). Moreover, YB1 has been reported to mediate drug resistance in multiple malignancies [16, 25], while EphA2, a member of the RTK family, has been reported to directly mediate resistance to RTK inhibitors in multiple malignancies [18, 19]. However, whether they mediate SUN resistance in kidney cancer remains unclear. GSEA analysis by using the ccRCC gene pool from TCGA database indicated that genes upregulated by high YB1 and EphA2 expression were enriched in the multiple-drug-resistance pathway, and genes upregulated by high EphA2 expression were enriched in renal cell-carcinoma pathway (Fig. 6a). We have previously successfully established SUN-resistant kidney cancer cell lines [26]. Western blot analysis revealed that the expression levels of YB1 and EphA2 were significantly higher in SUN-resistant cells than in the parental cells (Fig. 6b). Next, we examined YB1- and EphA2-mediated SUN sensitivity. Knockdown of YB1 increased the sensitivity of SUN-resistant cells to SUN (Fig. 6c). Similarly, knockdown of EphA2 also increased the sensitivity of SUN-resistant cells to SUN (Fig. 6d). Moreover, rescue experiments showed that the compromised resistance phenotype by EphA2 knockdown could be restored by the reintroduction of YB1 in SUN-resistant cells (Fig. 6e). These results indicated that YB1-mediated SUN resistance by modulating EphA2 signaling in human renal cancer cells.

### Pharmacological inhibition of EphA2 reduces the growth of SUN-resistant cells via the ERK/AKT/STAT3 signaling pathway in vitro

**Fig. 7:**
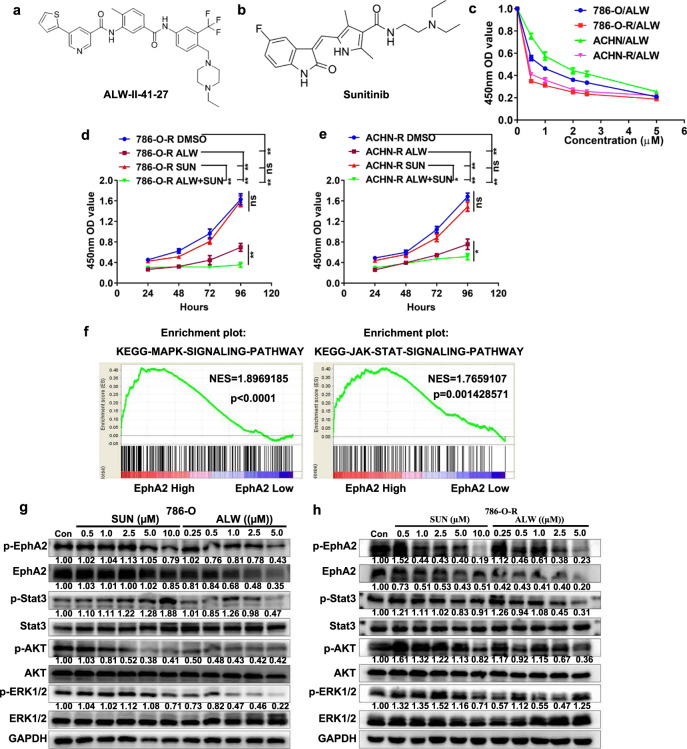
Pharmacological inhibition of EphA2 reduces the growth of sunitinib-resistant cells via the ERK/AKT/STAT3 signaling pathway in vitro. **a**, **b** Chemical structures of ALW-II-41-27 and sunitinib. **c** CCK8 assay analysis for the sensitivity of parent and sunitinib-resistant cells to ALW treatment. **d**, **e** CCK8 assay analysis of the sensitivity of 786-O-R and ACHN-R cells to treatment with DMSO, ALW alone, SUN alone, and ALW in combination with SUN. **f** GSEA analysis between EphA2 mRNA levels and MAPK and JAK/STAT signaling pathways. **g**, **h** Western blot analysis of EphA2, p-EphA2, Stat3, p-Stat3, AKT, p-AKT, ERK1/2, and p-ERK1/2 proteins after 786-O and 786-O-R cells were treated with different concentrations of SUN and ALW. The values indicate protein expression levels relative to GAPDH levels. (**P < 0.01, *P < 0.05, ns no significance, compared with the corresponding control).

Considering the potential roles of EphA2 in maintaining cell growth and in mediating SUN resistance, small molecule inhibitors of EphA2 could serve as a stepping stone for the transition from cell assays to clinical drug trials. To this end, we screened the EphA2 inhibitor ALW-II-41-27 (ALW) from the kinase inhibitor library. The chemical structures of ALW and SUN are shown in Fig. 7a, b. ALW effectively inhibited the growth of SUN-resistant and parental kidney cancer cells, and the inhibitory effect on SUN-resistant cells (high EphA2) was more obvious (Fig. 7c). Moreover, the combined treatment of ALW and SUN maximally inhibited the growth of SUN-resistant cells and enhanced the sensitivity of SUN-resistant cells to SUN (Fig. 7d, e). The combined treatment of ALW + SUN induced IC50 cell growth inhibition at a combination index of less than 0.7, indicating that ALW played a synergistic role with SUN in SUN-resistant cells (Supplementary Table 1). To explore the downstream signaling pathways involved in ALW inhibition of EphA2, GSEA analysis was used to examine signaling pathways enriched by EphA2-upregulated genes. As shown in Fig. 7f, genes upregulated by high EphA2 expression were enriched in the MAPK and JNK/STAT3 signaling pathways. To verify the results of the GSEA analysis, we used western blot to detect changes in protein expression in these signaling pathways after treatment with different concentrations of SUN or ALW. ALW treatment significantly inhibited the phosphorylation of EphA2, STAT3, AKT, and ERK1/2 in parental and SUN-resistant cells (Fig. 7g, h). It is well known that the ERK, AKT, and STAT3 signaling pathways play important roles in promoting cell growth and inhibiting apoptosis. Our results confirmed that ALW inhibited the activation and phosphorylation of these pathways, so we concluded that ALW suppressed the growth of parental and SUN-resistant cells by inhibiting these pathways.

### EphA2 mediates the growth and metastasis of SUN-resistant cells via the ERK/AKT/STAT3 signaling pathway in vivo

**Fig. 8:**
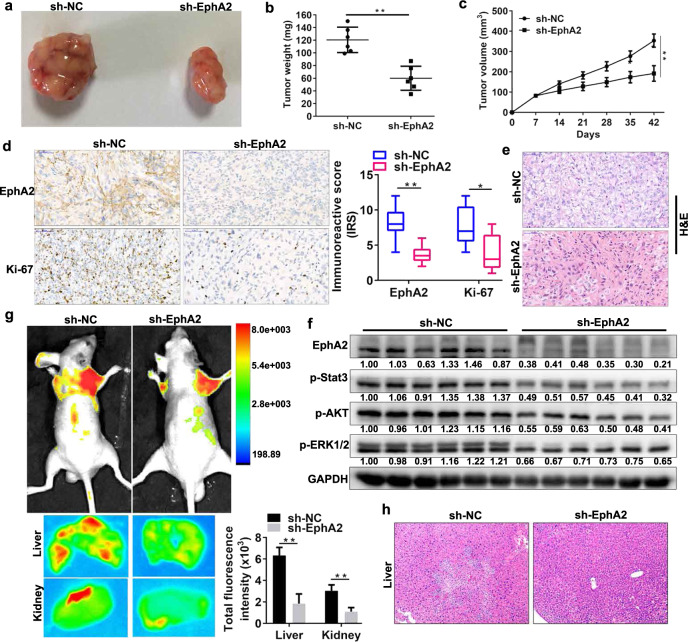
EphA2 mediates the growth and metastasis of sunitinib-resistant cells via the ERK/AKT/STAT3 signaling pathway in vivo. **a**–**c** The tumor volume was measured once a week. After 6 weeks, the mice were sacrificed, and the tumor weight was determined. EphA2 knockdown significantly reduced tumor size, weight and volume in 786-O-R tumors (*n* = 6 mice/group). **d** Immunohistochemical analysis of EphA2 and Ki-67 expression in the control and EphA2 knockdown tumor groups. The immunoreactive scores (IRS) of EphA2 and Ki-67 were presented. **e** H&E staining of tumor tissues in the control and EphA2 knockdown tumor groups. **f** Western blot analysis of EphA2, p-Stat3, p-AKT, and p-ERK1/2 proteins in the control and EphA2 knockdown tumor groups. The values indicate protein expression levels relative to GAPDH levels. **g** A total of 2 × 10^6^ cells expressing green fluorescent protein (GFP) were injected into the tail vein of BALB/c nude mice. In vivo live animal imaging and in vitro liver and kidney imaging analysis of tumor metastasis models in the control and EphA2 knockdown tumor groups (*n* = 6 mice/group). **h** H&E staining analysis of liver in the control and EphA2 knockdown tumor groups. (**P < 0.01, *P < 0.05, compared with the corresponding control).

To investigate the effect of EphA2 on the in vivo effects of SUN-resistant cells, we generated 786-O-R cells with stable EphA2 knockdown. We then injected these cells subcutaneously into nude mice to establish subcutaneous tumor models. We found that EphA2 knockdown significantly reduced tumor size, tumor weight, and tumor volume in the 786-O-R subcutaneous tumor model (Fig. 8a–c). IHC and haematoxylin and eosin (H&E) staining of subcutaneous tumor tissues demonstrated that EphA2 knockdown obviously decreased Ki-67 expression and tumor cell volume (Fig. 8d, e). Next, we explored changes in STAT3, AKT, and ERK pathways by western blot analysis in tumor tissues. The results showed that EphA2 knockdown inhibited the phosphorylation levels of the ERK/AKT/STAT3 pathway (Fig. 8f). In addition, we constructed a model of metastatic tumor in vivo via nude mouse tail vein injection of EphA2 knockdown 786-O-R cells. Small animal imaging system and H&E staining showed that EphA2 knockdown decreased the liver metastasis of tumor cells (Fig. 8g, h). These results indicate that EphA2 mediates the growth and metastasis of SUN-resistant cells via phosphorylation and activation of the ERK/AKT/STAT3 signaling pathway in vivo.

### Pharmacological inhibition of EphA2 decreases the growth of SUN-resistant tumors via the ERK/AKT/STAT3 signaling pathway in vivo

**Fig. 9:**
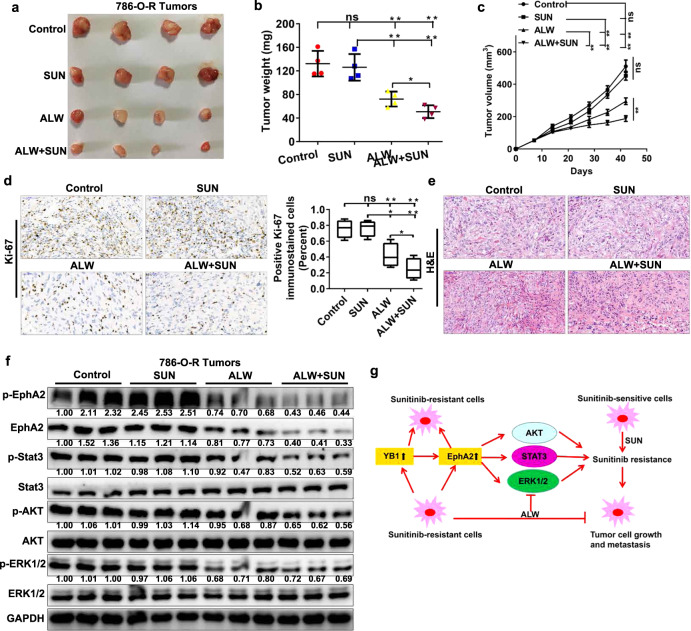
Pharmacological inhibition of EphA2 decreases the growth of sunitinib-resistant tumors via the ERK/AKT/STAT3 signaling pathway in vivo. **a**–**c** The tumor volume was measured once a week. After 6 weeks, the mice were sacrificed, and the tumor weight was determined. ALW alone or ALW combined with SUN treatment significantly reduced tumor size, weight and volume in 786-O-R tumors, while SUN alone did not (*n* = 4 mice/group). **d** Immunohistochemical analysis of Ki-67 expression in the control, ALW alone, SUN alone, and ALW combination with SUN treatment groups. The percentage of positive Ki-67 immunostained cells in each group was presented. **e** H&E staining analysis of tumor tissues in the control, ALW alone, SUN alone, and ALW combination with SUN treatment groups. **f** Western blot analysis of EphA2, p-EphA2, Stat3, p-Stat3, AKT, p-AKT, ERK1/2, and p-ERK1/2 proteins in the control, ALW alone, SUN alone, and ALW combination with SUN treatment groups. The values indicate protein expression levels relative to GAPDH levels. **g** Diagram of the YB1/EphA2 axis regulating tumor metastasis and sunitinib resistance (**P < 0.01, *P < 0.05, ns no significance, compared with the corresponding control).

Considering that the pharmacological inhibition of EphA2 significantly inhibited the growth of SUN-resistant cells in vitro and that the stable knockdown of EphA2 significantly decreased the growth of SUN-resistant tumors in vivo, we wondered whether the pharmacological inhibition of EphA2 could block the growth of SUN-resistant tumors in vivo. To evaluate the effects of ALW and ALW + SUN on tumors with acquired resistance to SUN in vivo, we treated subcutaneous xenografts (786-O-R tumors) with control, SUN, ALW, or ALW + SUN combination once every other day at 40 (SUN) or 15 (ALW) mg/kg via gavage administration. After the above treatment regimen was completed, ALW was found to significantly reduce the size and volume of SUN-resistant tumors (Fig. 9a–c). Moreover, the combined treatment of ALW and SUN reduced tumor size and volume more than ALW treatment alone (Fig. 9a–c), indicating that ALW treatment restored most, if not all, of the sensitivity of SUN-resistant tumors to SUN. Drug toxicity as measured by mouse body weight did not show a significant change in any drug treatment group or drug combination treatment group compared with the control group over the course of the study (data not shown). IHC and H&E staining of subcutaneous tumor tissues showed that combined treatment with ALW and SUN significantly reduced Ki-67 expression and tumor cell volume (Fig. 9d, e). Western blot analysis of tumor tissue lysates showed that ALW reduced the phosphorylation levels of EphA2, STAT3, AKT, and ERK (Fig. 9f), consistent with the results observed in vitro (Fig. 7g, h) and the results of stable knockdown of EphA2 observed in vivo (Fig. 8f). Based on our in vitro and in vivo findings, we mapped the mechanisms of RCC metastasis and SUN resistance mediated by the YB1/EphA2 axis (Fig. 9g). These findings indicate that pharmacological inhibition of EphA2 might be beneficial for SUN-resistant renal cancer, as inhibition of this protein could alleviate the phosphorylation and activation of key growth signaling pathway molecules and restore the sensitivity of SUN-resistant tumors to SUN.

## Discussion

Renal cancer, one of the most common malignancies of the urinary system, is characterized by frequent VHL gene inactivation and activation of the HIF–VEGF pathway, leading to extensive vascularization around kidney cancer. Extensive vascularization provides a favorable setting for the invasion and metastasis of RCC. RTK inhibitors such as SUN and sorafenib have been used clinically for the treatment of mRCC [3, 27]. However, intrinsic resistance and acquired resistance to RTK inhibitors greatly reduce the therapeutic efficiency of these inhibitors. Therefore, the metastasis and RTK inhibitor resistance of renal cancer urgently need to be resolved. However, the specific molecular mechanisms of renal cancer metastasis and RTK inhibitor resistance are still unknown.

YB1, as a DNA and RNA-binding protein, promotes the metastasis of multiple tumors by transcriptionally or translationally activating the expression of various metastasis-associated proteins [12–15]. Specifically, YB1 translationally activates HIF1α expression to promote sarcoma metastasis and activates Snail1 and other transcription factors to induce EMT in breast cancer [14]. EphA2, a member of the RTK family, has been reported by us and other study groups to promote the dissemination and metastasis of tumors [18, 22]. Moreover, both YB1 and EphA2 have been reported to promote cell metastasis of renal cancer in vitro [11, 22]. However, the link between YB1 and EphA2 has not yet been established.

Cancer drug resistance has always been the focus and difficulty of clinical treatment. Therefore, drug resistance studies have always been a hot spot for oncologists. Our previous results indicated that TR4 nuclear receptor-mediated lncTASR/AXL signaling promoted RCC SUN resistance, and this effect could be reversed by tretinoin [28]. YB1 transcriptionally regulates the expression of multiple-drug resistance-associated genes to mediate multidrug resistance in a variety of tumors [29]. EphA2 has also been reported to mediate resistance against multiple RTK inhibitors in various malignancies. For example, EphA2 mediates EGFR kinase inhibitor resistance in lung cancer and mediates vemurafenib resistance in melanoma [18, 19]. Consistent with our results, previous literature has reported that YB1 mediates RCC SUN resistance [30]. However, the roles of EphA2 and its association with YB1 in SUN resistance have not been reported in renal cancer. Our results suggest that the expression levels of YB1 and EphA2 are upregulated in SUN-resistant cells and that their high expression levels mediate the resistance of renal cancer cells to SUN. Knockdown of YB1 and EphA2 increases the sensitivity of SUN-resistant kidney cancer cells to SUN treatment. Moreover, YB1-promoted SUN resistance is mediated by EphA2. EphA2 knockdown by shRNA counteracts YB1-mediated renal cancer SUN resistance in vitro, and this effect is also confirmed by EphA2 small molecule inhibitor ALW. These results render YB1 and EphA2 ideal targets for treatment in SUN-resistant renal cancer patients.

In conclusion, our findings indicate that YB1 and EphA2 levels are highly expressed in renal cancer cells and tissues and further increase in SUN-resistant cells. Functionally, high YB1 and EphA2 expression levels promote the invasion, metastasis, and SUN resistance of RCC. Mechanistically, YB1 increases the protein expression of EphA2 by maintaining its protein stabilization. These results provide a new and promising strategy for the treatment of patients with renal cancer who already have distant metastases or who have developed resistance against SUN.

## Materials and methods

Cell transfection, cell infection, reagents, plasmid construction, immunoprecipitation (IP), western blotting assays, and bioinformatic analysis are described in detail in Supplementary Methods and Materials.

### Renal cancer tissue samples

Human renal cancer TMA was purchased from Wuhan Servicebio Co., Ltd (catalog: RC-1501). The TMA contained detailed clinical and pathological information.

We collected 50 pairs of ccRCC and normal adjacent tissues at Wuhan Union Hospital between 2016 and 2018. Isolated tissues were immediately stored in liquid nitrogen. None of the patients had received any radiotherapy, chemotherapy, immunotherapy, or targeted therapies before surgery. All patients signed the informed consent form. All experimental procedures were approved by the Human Ethics Committee of Huazhong University of Science and Technology.

### Cell culture

The human renal cancer cell lines 786-O, ACHN, and Caki-1 and the normal renal epithelial cell line HK-2 were purchased from ATCC. SUN-resistant cell lines (786-O-R and ACHN-R) were induced and established in our laboratory [26]. Cell culture was performed as previously described [26].

### IHC and immunofluorescence (IF) assays

IHC analysis of human renal cancer tissues was performed using a monoclonal EphA2 antibody (sc-398832, Santa Cruz) and a polyclonal YB1 antibody (20339-1-AP, Proteintech, China). Specific assays were performed as previously described [31]. The immunoreactive score of the IHC results was then scored by two independent pathologists blinded to the tissue specimen information [16]. *H*-scores of YB1 protein immunohistochemical staining were analyzed and evaluated as described previously [32]. For the IF experiments, cells were fixed in 4% paraformaldehyde, permeabilized with 0.3% Triton X-100, blocked with 3% BSA for 1 h at 37 °C, and then incubated with primary antibody and fluorescent secondary antibodies.

### ChIP assays

ChIP assays were performed as previously described [33]. The primer sequences for the EphA2 promoter region were as follows:

P1 (−66 to −215): 3′-TACCAGGCTCAGAGATCCCT-5′, 3′-GGAGGAGAGGGAGGGGAAG-5′;

P2 (−260 to −425): 3′-AGGGTTTTAGGAAGGAGGGC-5′, 3′-TTCTGCCCTTCACCTCTGAG-5′;

P3 (−621 to −798): 3′-TAGGCAGGTGTCCTCCAAAC-5′, 3′-GAGCAGAAACAACATGCCCA-5′;

P4 (−870 to −1019): 3′-CAAAGGGAGTCTGGGGCC-5′, 3′-GCGGCTAGTGTGAGTGAAAC-5′;

P5 (−1268 to −1417): 3′-CACCCTGCCCAGTCACATA-5′, 3′-AAGTGCCTGTGAGAATAAGTGC-5′;

P6 (−1535 to −1684): 3′-ACAGAGTCTTGCTGGCCC-5′, 3′-AAAATTAGCTGGGCGTGGTG-5′.

Monoclonal YB1 antibody (sc-101198, Santa Cruz Biotechnology) was used for IP, and normal IgG served as a negative control. We retained 2% of the original DNA as a positive input control.

### Microarray analysis

786-O cells with YB1 knockdown or vector were harvested at 7 days after puromycin selection. Microarray analysis was performed with three duplicate samples by Shanghai OE Biotech Co., Ltd. Differentially expressed genes between 786-O shYB1 and 786-O vector cells were selected based on a mean fold change of 2 and a *P* value < 0.05. The RNA-Seq data have been deposited to GEO under the accession number GSE 151336.

### Wound healing assays and transwell assays

Wound healing assays and transwell assays were performed as previously described [31].

### Quantitative real-time PCR assays (qRT-PCR)

qRT-PCR was performed as previously described [31].

### In vivo RCC subcutaneous and metastatic tumor models

A total of 2 × 10^6^ cells expressing green fluorescent protein were injected into the tail vein of BALB/c nude mice purchased from Beijing HFK Bio-technology. Tumor metastatic lesions were measured using a live animal imaging system. After the nude mice were sacrificed at 6 weeks, the metastatic lesions were stained with H&E. For the RCC subcutaneous tumor model, the experimental procedures were performed as described previously [16]. The tumor volume was measured once a week. After 6 weeks, the mice were sacrificed and the tumor weight was measured. SUN was orally administered via gavage needle at 40 mg/kg every other day, and ALW was orally administered via gavage needle at 15 mg/kg every other day. All animal experiments were approved by the Animal Ethics Committee of Tongji Medical College of Huazhong University of Science and Technology.

### Statistical analysis

The statistical analysis was performed using SPSS statistical software 22.0 (IBM SPSS, USA) or GraphPad Prism 7.0 (GraphPad software, Inc., USA). Data are presented as the mean ± SEM. The error bars indicate the mean ± SEM of three independent assays. Statistical analyses were performed using the Mann–Whitney test and Student’s *t* test and the Pearson correlation coefficient. The Kaplan–Meier curve and log-rank test were used to evaluate the survival of patients. Statistical significance was determined when the *P* value was less than 0.05.

## Supplementary information

Supplemental Material

## References

[1] Gupta GP, Massague J (2006). Cancer metastasis: building a framework. Cell.

[2] Cutz JC, Guan J, Bayani J, Yoshimoto M, Xue H, Sutcliffe M (2006). Establishment in severe combined immunodeficiency mice of subrenal capsule xenografts and transplantable tumor lines from a variety of primary human lung cancers: potential models for studying tumor progression-related changes. Clin Cancer Res.

[3] Motzer RJ, Hutson TE, Tomczak P, Michaelson MD, Bukowski RM, Rixe O (2007). Sunitinib versus interferon alfa in metastatic renal-cell carcinoma. N Engl J Med.

[4] Motzer RJ, Hutson TE, Tomczak P, Michaelson MD, Bukowski RM, Oudard S (2009). Overall survival and updated results for sunitinib compared with interferon alfa in patients with metastatic renal cell carcinoma. J Clin Oncol.

[5] Park K, Lee JL, Park I, Park S, Ahn Y, Ahn JH (2012). Comparative efficacy of vascular endothelial growth factor (VEGF) tyrosine kinase inhibitor (TKI) and mammalian target of rapamycin (mTOR) inhibitor as second-line therapy in patients with metastatic renal cell carcinoma after the failure of first-line VEGF TKI. Med Oncol.

[6] Gotink KJ, Broxterman HJ, Labots M, de Haas RR, Dekker H, Honeywell RJ (2011). Lysosomal sequestration of sunitinib: a novel mechanism of drug resistance. Clin Cancer Res.

[7] Joosten SC, Hamming L, Soetekouw PM, Aarts MJ, Veeck J, van Engeland M (2015). Resistance to sunitinib in renal cell carcinoma: from molecular mechanisms to predictive markers and future perspectives. Biochim Biophys Acta.

[8] Lim JP, Nair S, Shyamasundar S, Chua PJ, Muniasamy U, Matsumoto K (2019). Silencing Y-box binding protein-1 inhibits triple-negative breast cancer cell invasiveness via regulation of MMP1 and beta-catenin expression. Cancer Lett.

[9] Kosnopfel C, Sinnberg T, Sauer B, Busch C, Niessner H, Schmitt A (2018). YB-1 expression and phosphorylation regulate tumorigenicity and invasiveness in melanoma by influencing EMT. Mol Cancer Res.

[10] Miao X, Wu Y, Wang Y, Zhu X, Yin H, He Y (2016). Y-box-binding protein-1 (YB-1) promotes cell proliferation, adhesion and drug resistance in diffuse large B-cell lymphoma. Exp Cell Res.

[11] Wang Y, Chen Y, Geng H, Qi C, Liu Y, Yue D (2015). Overexpression of YB1 and EZH2 are associated with cancer metastasis and poor prognosis in renal cell carcinomas. Tumour Biol.

[12] Jurchott K, Bergmann S, Stein U, Walther W, Janz M, Manni I (2003). YB-1 as a cell cycle-regulated transcription factor facilitating cyclin A and cyclin B1 gene expression. J Biol Chem.

[13] Shibao Kazunori, HT, Nakayama Yoshifumi, Okazaki Keisuke, Nagata Naoki, Izumi Hiroto (1999). Enhanced coexpression of YB-1 and DNA topoisomerase II alpha genes in human colorectal carcinomas. Int J Cancer.

[14] Evdokimova V, Tognon C, Ng T, Ruzanov P, Melnyk N, Fink D (2009). Translational activation of snail1 and other developmentally regulated transcription factors by YB-1 promotes an epithelial-mesenchymal transition. Cancer Cell.

[15] El-Naggar AM, Veinotte CJ, Cheng H, Grunewald TG, Negri GL, Somasekharan SP (2015). Translational activation of HIF1alpha by YB-1 promotes sarcoma metastasis. Cancer Cell.

[16] Tao Z, Ruan H, Sun L, Kuang D, Song Y, Wang Q (2019). Targeting the YB-1/PD-L1 axis to enhance chemotherapy and antitumor immunity. Cancer Immunol Res.

[17] Chu PC, Lin PC, Wu HY, Lin KT, Wu C, Bekaii-Saab T (2018). Mutant KRAS promotes liver metastasis of colorectal cancer, in part, by upregulating the MEK-Sp1-DNMT1-miR-137-YB-1-IGF-IR signaling pathway. Oncogene.

[18] Miao B, Ji Z, Tan L, Taylor M, Zhang J, Choi HG (2015). EPHA2 is a mediator of vemurafenib resistance and a novel therapeutic target in melanoma. Cancer Discov.

[19] Amato KR, Wang S, Tan L, Hastings AK, Song W, Lovly CM (2016). EPHA2 blockade overcomes acquired resistance to EGFR kinase inhibitors in lung cancer. Cancer Res.

[20] Miyazaki T, Kato H, Fukuchi M, Nakajima M, Kuwano H (2003). EphA2 overexpression correlates with poor prognosis in esophageal squamous cell carcinoma. Int J Cancer.

[21] Liu F, Park PJ, Lai W, Maher E, Chakravarti A, Durso L (2006). A genome-wide screen reveals functional gene clusters in the cancer genome and identifies EphA2 as a mitogen in glioblastoma. Cancer Res.

[22] Chen X, Wang X, Ruan A, Han W, Zhao Y, Lu X (2014). miR-141 is a key regulator of renal cell carcinoma proliferation and metastasis by controlling EphA2 expression. Clin Cancer Res.

[23] Herrem CJ, Tatsumi T, Olson KS, Shirai K, Finke JH, Bukowski RM (2005). Expression of EphA2 is prognostic of disease-free interval and overall survival in surgically treated patients with renal cell carcinoma. Clin Cancer Res.

[24] Ruan H, Song Z, Cao Q, Ni D, Xu T, Wang K (2020). IMPDH1/YB-1 positive feedback loop assembles cytoophidia and represents a therapeutic target in metastatic tumors. Mol Ther.

[25] Shiota M, Fujimoto N, Imada K, Yokomizo A, Itsumi M, Takeuchi A, et al. Potential role for YB-1 in castration-resistant prostate cancer and resistance to enzalutamide through the androgen receptor V7. J Natl Cancer Inst. 2016;108. 10.1093/jnci/djw005. 10.1093/jnci/djw005 26857528

[26] Ruan H, Li X, Yang H, Song Z, Tong J, Cao Q (2017). Enhanced expression of caveolin-1 possesses diagnostic and prognostic value and promotes cell migration, invasion and sunitinib resistance in the clear cell renal cell carcinoma. Exp Cell Res.

[27] Escudier Bernard, Eisen Tim, Stadler WalterM, Szczylik Cezary, Oudard Stéphane, Siebels Michael (2007). Sorafenib in advanced clear-cell renal-cell carcinoma. N Engl J Med.

[28] Shi H, Sun Y, He M, Yang X, Hamada M, Fukunaga T (2020). Targeting the TR4 nuclear receptor-mediated lncTASR/AXL signaling with tretinoin increases the sunitinib sensitivity to better suppress the RCC progression. Oncogene.

[29] Kuwano M, Shibata T, Watari K, Ono M (2019). Oncogenic Y-box binding protein-1 as an effective therapeutic target in drug-resistant cancer. Cancer Sci.

[30] D’Costa NM, Lowerison MR, Raven PA, Tan Z, Roberts ME, Shrestha R (2020). Y-box binding protein-1 is crucial in acquired drug resistance development in metastatic clear-cell renal cell carcinoma. J Exp Clin Cancer Res.

[31] Ruan H, Yang H, Wei H, Xiao W, Lou N, Qiu B (2017). Overexpression of SOX4 promotes cell migration and invasion of renal cell carcinoma by inducing epithelial-mesenchymal transition. Int J Oncol.

[32] Yang Y, Xiao M, Song Y, Tang Y, Luo T, Yang S (2019). H-score of 11beta-hydroxylase and aldosterone synthase in the histopathological diagnosis of adrenocortical tumors. Endocrine.

[33] Yamashita T, Higashi M, Momose S, Morozumi M, Tamaru JI (2017). Nuclear expression of Y box binding-1 is important for resistance to chemotherapy including gemcitabine in TP53-mutated bladder cancer. Int J Oncol.

