# Correction: Enhanced YB1/EphA2 axis signaling promotes acquired resistance to sunitinib and metastatic potential in renal cell carcinoma

**DOI:** 10.1038/s41388-022-02534-0

**Published:** 2022-11-30

**Authors:** Hailong Ruan, Sen Li, Lin Bao, Xiaoping Zhang

**Affiliations:** 1 Department of Urology, Union Hospital, Tongji Medical College, Huazhong University of Science and Technology, Wuhan, 430022 China; 2 Institute of Urology, Union Hospital, Tongji Medical College, Huazhong University of Science and Technology, Wuhan, 430022 China

**Keywords:** Renal cell carcinoma, Cell migration

Correction to: *Oncogene* 10.1038/s41388-020-01409-6, published online 19 August 2020

Following the publication of this article, the authors noted incorrect transwell images included in Fig. 2h-i, Fig. 4f-h and Supplementary Fig. 2d. The authors confirm these inadvertent errors do not affect the conclusions presented in the article and apologize for any inconvenience caused.
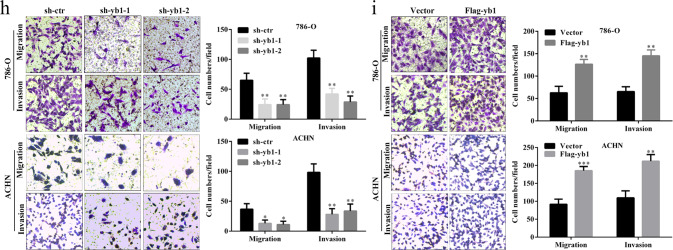

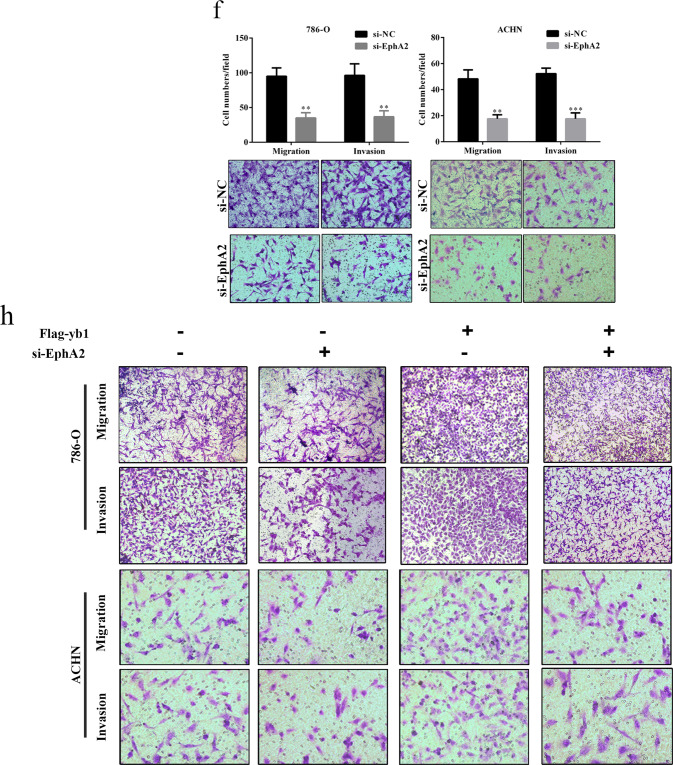


The original article has been corrected.

## Supplementary information

Supplementary figure

