# Supplementary material for: Enhanced YB1/EphA2 axis signaling promotes acquired resistance to sunitinib and metastatic potential in renal cell carcinoma
Source: Oncogene. 2020 Aug 19;39(38):6113–28. doi: 10.1038/s41388-020-01409-6 (PMC7498371; doi:10.1038/s41388-020-01409-6)
Supplement: Supplementary file 1 — Supplemental Material [file 41388_2020_1409_MOESM1_ESM.docx]

**Enhanced YB1/EphA2 axis signaling promotes acquired resistance to sunitinib and metastatic potential in renal cell carcinoma**

Hailong Ruan^1,2*^, Sen Li ^1,2^, Lin Bao^1,2*^, Xiaoping Zhang^1,2*^

^1^Department of Urology, Union Hospital, Tongji Medical College, Huazhong University of Science and Technology, Wuhan 430022, China; ^2^Institute of Urology, Union Hospital, Tongji Medical College, Huazhong University of Science and Technology, Wuhan 430022, China;

Running Head: YB1/EphA2 blockade overcomes metastasis and sunitinib resistance in RCC

*Corresponding author:

Hailong Ruan, E-mail: [hlruan2018@hust.edu.cn](mailto:hlruan2018@hust.edu.cn); Lin Bao, E-mail: [2417221775@qq.com](mailto:2417221775@qq.com); Xiaoping Zhang, E-mail: [xzhang@hust.edu.cn](mailto:xzhang@hust.edu.cn)

**Supplementary methods and materials**

**Cell transfection, infection, reagents and plasmid construction**

Small hairpin RNAs (shRNAs) against EphA2 (sh-EphA2), and YB1 (sh-YB1) and the corresponding blank control with nonsense sequence were purchased from Shanghai GENECHEM. The pGV341-FLAG-YB1 construct (YB1) and empty vector (Vector) were purchased from Shanghai GENECHEM. The lentiviral particles containing pGV341-FLAG-YB1, pGV248-sh-YB1 and pGV248-sh-EphA2 were purchased from Shanghai GENECHEM and used to infect RCC cells according to the manufacturer's recommendations. Small interfering RNA against EphA2 (si-EphA2) and the corresponding control (si-NC) were purchased from Guangzhou RiboBio. Plasmids were transfected using Lipofectamine 2000 according to the manufacturer's recommendations. ALW-II-41-27 (ALW) and sunitinib (SUN) were purchased from MedChemExpress (MCE, USA). MG-132 and cycloheximide (CHX) were purchased from MedChemExpress (MCE, USA).

**Immunoprecipitation (IP) and western blotting assays**

Cells were resuspended and lysed in RIPA buffer containing the protease inhibitors PMSF and cocktail. We retained 2% of the lysates as input, and the remaining lysates were used for IP. Antibodies for western blotting and IP were as follows: anti-EphA2 (sc-398832, Santa Cruz Biotechnology), anti-YB1 (sc-101198, Santa Cruz Biotechnology), anti-p-EphA2 (AP1082, ABclonal Technology), anti-Ubiquitin (A19686, ABclonal Technology), anti-STAT3 ([sc-8019](https://www.scbt.com/zh/p/stat3-antibody-f-2?requestFrom=search), Santa Cruz Biotechnology), anti-p-STAT3 ([sc-8059](https://www.scbt.com/zh/p/stat3-antibody-f-2?requestFrom=search), Santa Cruz Biotechnology), anti-AKT (sc-5298, Santa Cruz Biotechnology), anti-p-AKT (sc-293125, Santa Cruz Biotechnology), anti-ERK1/2 (sc-135900, Santa Cruz Biotechnology), anti-p-ERK1/2 (sc-81492, Santa Cruz Biotechnology), anti-Actin (sc-58673, Santa Cruz Biotechnology), anti-FLAG (66008-2-Ig, 20543-1-AP, Proteintech, China), and anti-GAPDH (sc-47724, Santa Cruz Biotechnology).

**Bioinformatic analysis**

Standardized mRNA expression data for RCC were downloaded from TCGA database and used to evaluate the expression and correlation of EphA2, YB1, E-cadherin, N-cadherin, Occludin, Vimentin, Snail1, Snail2, MMP2, MMP9, Fibronectin, Twist1 and Twist2. These data were also used to assess overall survival (OS) and disease-free survival (DFS) in RCC patients. Spearman's correlation coefficient was calculated for the mRNA levels for RCC samples. P < 0.05 was considered statistically significant.

**Supplementary Table 1.** ALW + SUN combination index (CI) leading to a 50% inhibition of cell growth for sunitinib-resistant cell lines

| Cell lines | IC50 drug alone | | IC50 drug combination | | | CI at IC50^*^ |
| --- | --- | --- | --- | --- | --- | --- |
|  | ALW (μM) | SUN (μM) | | ALW (μM) | SUN (μM) |  |
| 786-O-R | 0.39 | 20.22 | | 0.16 | 5.25 | 0.67 |
| ACHN-R | 0.43 | 24.50 | | 0.19 | 6.17 | 0.69 |

*Combination index (CI) was calculated at IC50 based on Chou-Talalay method


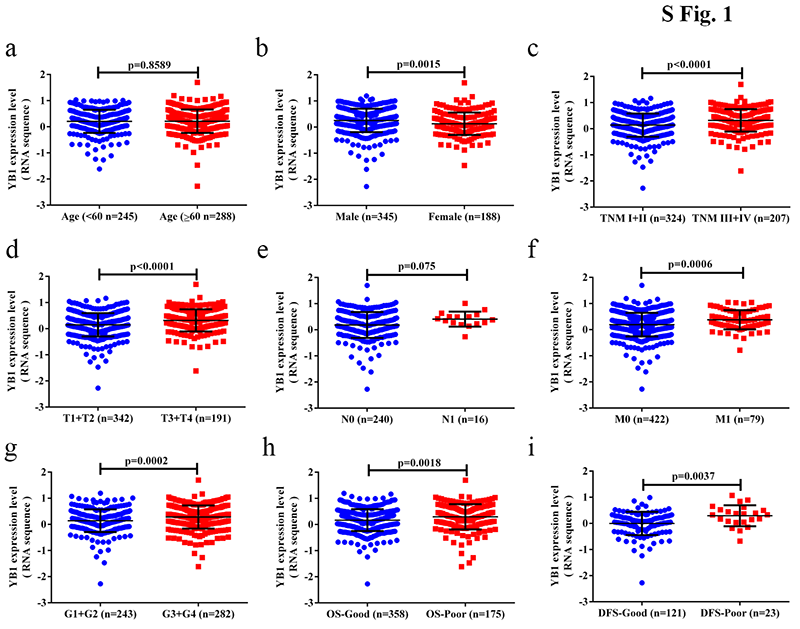


**Supplementary Figure 1. The expression of YB1 correlates with a wide range of clinicopathological parameters in ccRCC.** (a-i) The mRNA level of YB1 in ccRCC was downloaded from the TCGA_KIRC dataset composed of 534 ccRCC tissues, including 72 paired tissues. The mRNA levels of YB1 were compared in different clinicopathological parameters: (a) age, (b) gender, (c) TNM stage, (d) T stage, (e) N stage, (f) M stage, (g) G stage, (h) OS, (i) DFS.
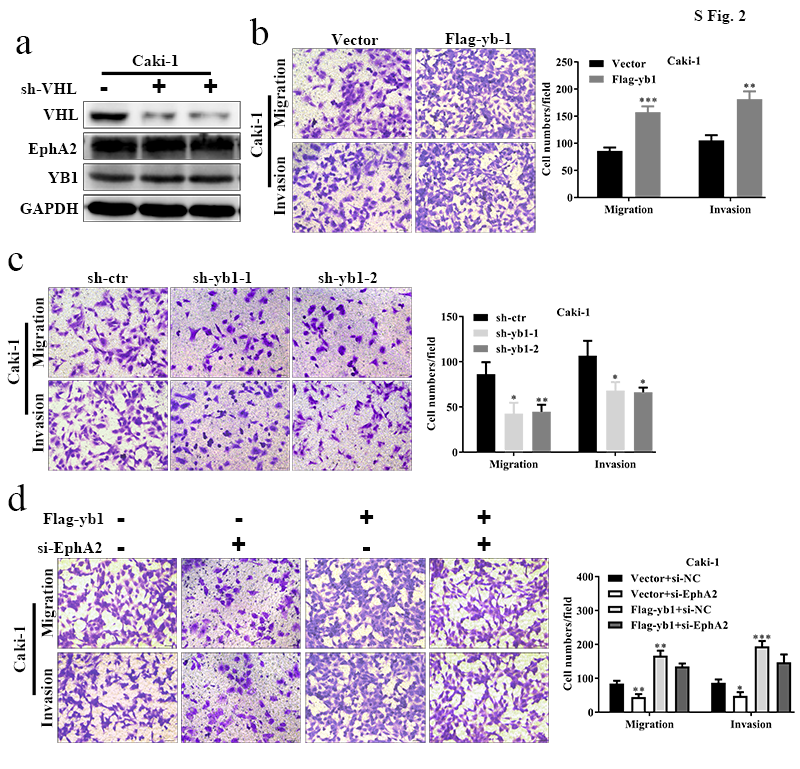
 **Supplementary Figure 2. Loss of VHL has no effect on the YB1/EphA2 axis and the phenotype induced by YB1/EphA2 axis.** (a) Western blotting analysis of EphA2 and YB1 expression in VHL-knockdown Caki-1 cells. (b-c) Transwell experimental analysis of the effect of stable YB1 overexpression or knockdown on cell migration and invasion in Caki-1 cells. (d) Transwell analysis of cell migration and invasion capability in Caki-1 cells with YB1 overexpression/EphA2 knockdown or Vector/EphA2 knockdown (***, P < 0.001, **, P < 0.01, *, P < 0.05, compared with the corresponding control).


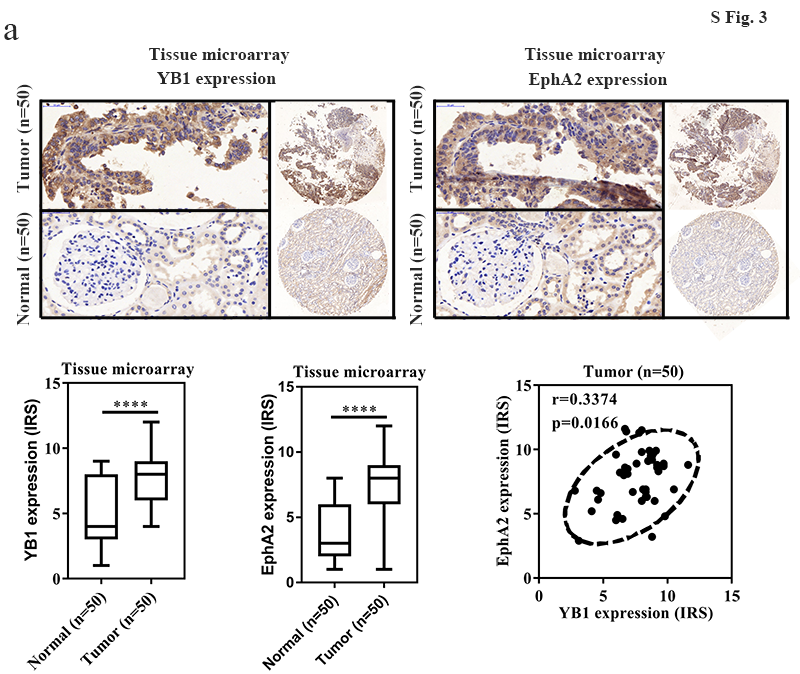


**Supplementary Figure 3. TMA immunohistochemical staining of YB1 and EphA2 proteins.** (a) Tissue microarray (TMA) is composed of 50 pairs of ccRCC tissues (Normal and Tumor), stained with YB1 antibody and EphA2 antibody, respectively. The expression of YB1 and EphA2 proteins was quantified using immunooreactive score (IRS) by 2 independent pathologists blinded to the TMA information (****, P<0.0001, compared with the corresponding control).


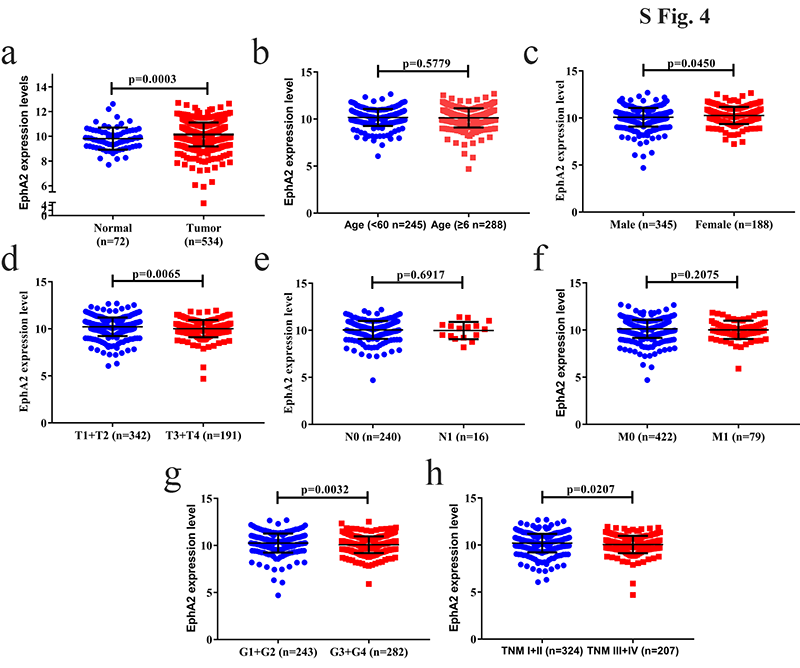
 **Supplementary Figure 4. The expression of EphA2 correlates with a wide range of clinicopathological parameters in ccRCC.** (a-h) The mRNA level of EphA2 in ccRCC was downloaded from the TCGA_KIRC dataset composed of 534 ccRCC tissues, including 72 paired tissues. The mRNA levels of EphA2 were compared in different clinicopathological parameters: (a) Normal versus Tumor, (b) age, (c) gender, (d) T stage, (e) N stage, (f) M stage, (g) G stage, (h) TNM stage.


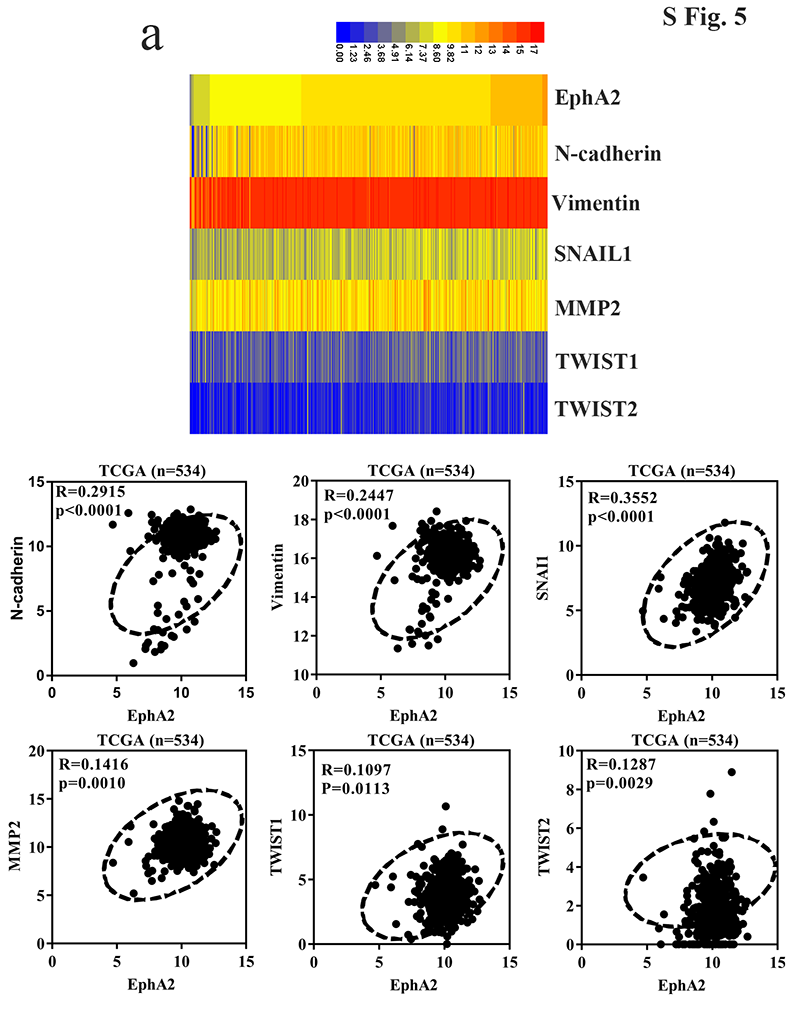
 **Supplementary Figure 5. Correlation analysis between EphA2 and EMT markers.** (a) Heat map and correlation analysis depicting the association of EphA2 with EMT markers.
