# Supplementary figures and images for: Correction: Enhanced YB1/EphA2 axis signaling promotes acquired resistance to sunitinib and metastatic potential in renal cell carcinoma
Source: Oncogene. 2022 Nov 30;42(2):165–7. doi: 10.1038/s41388-022-02534-0 (PMC9816057; doi:10.1038/s41388-022-02534-0)

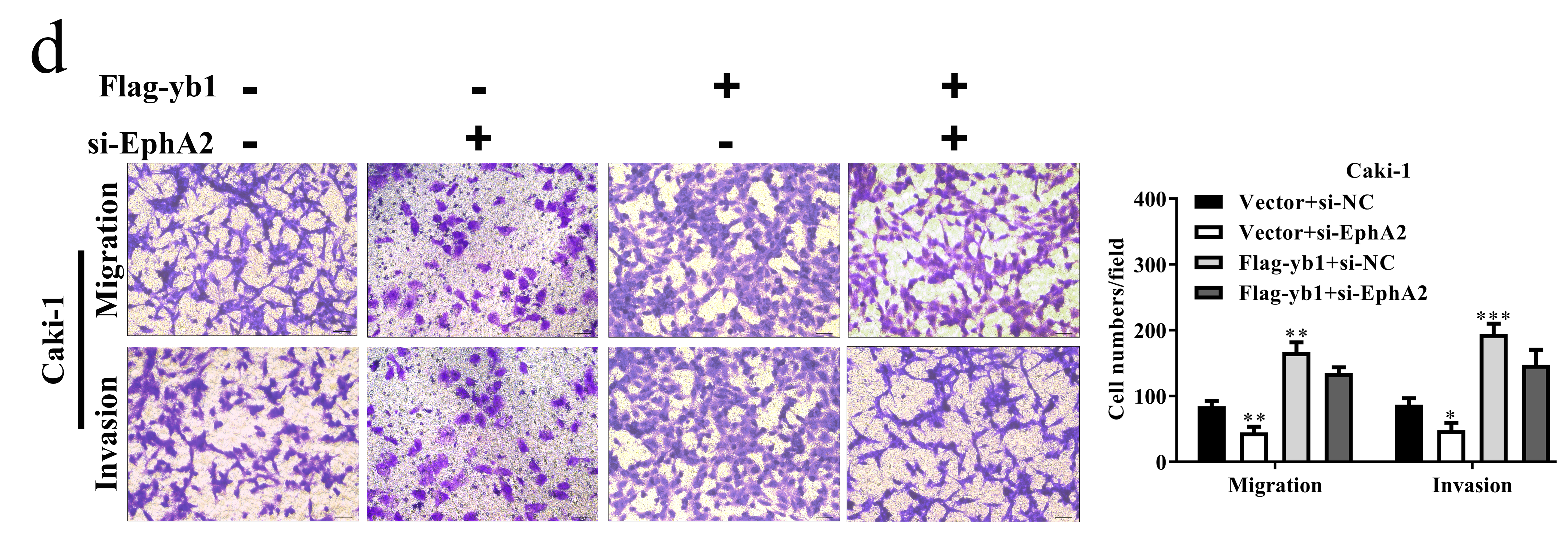

Supplement: Supplementary file 1 — Supplementary figure [file 41388_2022_2534_MOESM1_ESM.tif]
